# New Perspectives on the Molecular Action of Metformin in the Context of Cellular Transduction and Adipogenesis

**DOI:** 10.3390/ijms26083690

**Published:** 2025-04-14

**Authors:** Jorge Enrique González-Casanova, Mario Navarro-Marquez, Tamara Saez-Tamayo, Lissé Angarita, Samuel Durán-Agüero, Héctor Fuentes-Barría, Valmore Bermúdez, Diana Marcela Rojas-Gómez

**Affiliations:** 1 Facultad de Ciencias de la Salud, Instituto de Ciencias Biomédicas, Universidad Autónoma de Chile, Santiago 8910060, Chile; jorge.gonzalez@uautonoma.cl; 2 Escuela de Química y Farmacia, Facultad de Medicina, Universidad Andres Bello, Santiago 8370321, Chile; mario.navarro@unab.cl (M.N.-M.); t.seztamayo@uandresbello.edu (T.S.-T.); 3 Escuela de Nutrición y Dietética, Facultad de Medicina, Universidad Andres Bello, Concepción 4260000, Chile; lisse.angarita@unab.cl; 4 Escuela de Nutrición y Dietética, Facultad de Ciencias de la Rehabilitación y Calidad de Vida, Universidad San Sebastián, Sede Los Leones, Lota 2465, Providencia, Santiago 7500000, Chile; samuel.duran@uss.cl; 5 Vicerrectoría de Investigación e Innovación, Universidad Arturo Prat, Iquique 1100000, Chile; hectorfuentesbarria@gmail.com; 6 Facultad de Ciencias de la Salud, Centro de Investigaciones en Ciencias de la vida, Universidad Simón Bolívar, Barranquilla 080022, Colombia; 7 Escuela de Nutrición y Dietética, Facultad de Medicina, Universidad Andres Bello, Santiago 8370321, Chile

**Keywords:** adipogenesis, metformin, AMPK, vitamin D

## Abstract

Metformin, a widely used antidiabetic drug, modulates the cellular physiology and metabolism of various body tissues, including adipose tissue. Adipogenesis, a complex process in which mesenchymal stem cells (MSC) differentiate into functional adipocytes, plays a key role in metabolic health and represents a potential therapeutic target for diverse metabolic disorders. Notably, recent evidence suggests that metformin modulates adipocyte differentiation. This narrative review explores the effects of metformin on cellular metabolism, with a particular focus on adipogenesis. The findings compiled in this review show that metformin regulates glucose and lipid metabolism in multiple tissues, including skeletal muscle, adipose tissue, liver, and intestine. Furthermore, metformin modulates adipogenesis through AMP-activated protein kinase (AMPK)-dependent and independent mechanisms in 3T3-L1 cells and adipose-derived stem cells. The review also emphasizes that metformin can promote or inhibit adipogenesis and lipid accumulation, depending on its concentration. Additionally, metformin attenuates inflammatory pathways by reducing the production of proinflammatory cytokines such as IL-6, MCP-1, and COX-2. Finally, evidence supports that vitamin D enhances the anti-inflammatory actions of metformin and promotes cell differentiation toward a beige adipocyte phenotype. In summary, this review examines the molecular actions of metformin to propose potential new therapeutic strategies for managing obesity and related metabolic diseases.

## 1. Introduction

Type 2 diabetes mellitus (T2DM) and obesity are two of the major chronic non-communicable diseases that represent a significant global public health challenge. T2DM is characterised by chronic hyperglycaemia due to a combination of insulin resistance and insufficient insulin secretion from the β-cells of the pancreatic islets [1,2]. The prevalence of these diseases has risen alarmingly in recent decades, mainly due to unhealthy dietary patterns, sedentary lifestyles, and an aging population. It is estimated that more than 537 million adults will be living with diabetes in 2021, and this number is projected to reach 783 million by 2045 [3].

In 1957, Jean Sterne introduced metformin as a therapeutic option for T2DM, revolutionising the management of this chronic condition. The clinical benefits of metformin are strongly supported by evidence, including its long-term safety and efficacy, low risk of hypoglycaemia, and positive effects on cardiovascular health and mortality [4,5]. In addition, its therapeutic profile includes advantages such as moderate body weight reduction, low cost, and wide availability, making it an accessible and effective option [6]. More than 200 million people with T2DM worldwide use metformin as part of their daily treatment. It is used as monotherapy and with other antidiabetic agents, such as sulphonylureas or dipeptidyl peptidase-4 (DPP-4) inhibitors, showing additive or synergistic effects optimizing glycemic control. These characteristics consolidate metformin as a central pillar in the treatment of T2DM. Surprisingly, the mechanisms underlying its therapeutic action are complex and still not fully understood.

The effect of metformin on the AMPK pathway and other molecular mechanisms offers promising prospects for understanding and treating metabolic disorders. In this context, metformin has shown a significant impact on adipogenesis. Several experimental studies have suggested that metformin promotes triglyceride catabolism in adipocytes by activating AMP-activated protein kinase (AMPK) and inhibits lipid accumulation in preadipocytes, thereby reducing fat storage.

Adipogenesis is the process of cell differentiation that transforms mesenchymal stem cells into mature adipocytes, characterised by their ability to store lipids and regulate energy metabolism. This process occurs in several well-defined stages and is regulated by key transcription factors such as peroxisome proliferator-activated receptor gamma (PPARγ) and CCAAT/enhancer-binding proteins (C/EBPβ and C/EBPα). These factors, especially PPARγ, play a key role in adipocyte maturation and are associated with metabolic pathologies such as obesity and insulin resistance [7]. This article reviews recent findings on the effects of metformin on adipogenesis and its potential impact on metabolic health, focusing on its therapeutic applications in obesity and related disorders.

## 2. Metabolic Actions of Metformin: Mechanisms and Effects Across Key Tissues

Metformin, a drug from the biguanide family, is primarily prescribed for managing T2DM, especially in individuals with overweight or obesity, due to its metabolic benefits beyond glucose-lowering effects [6]. Additionally, it has been linked to several off-label uses, including the treatment of polycystic ovary syndrome, steatohepatitis, and metabolic disorders associated with HIV [8].

Metformin is administered orally and is available in immediate and extended-release formulations [9]. With a pKa of 12.3, metformin predominantly exists in a hydrophilic, positively charged form at physiological pH, a key factor influencing its pharmacokinetics [10]. This charge minimizes its ability to dissolve in lipids, reducing the likelihood of passive diffusion across cell membranes. As a result, metformin depends on specific transport proteins for its pharmacokinetics. PMAT (plasma membrane monoamine transporter) facilitates the absorption of metformin in the gastrointestinal tract. In contrast, OCT1 and OCT3 (organic cation transporters 1 and 3) are crucial for its distribution to the liver and its excretion in an unchanged form through the kidneys [9].

The most common adverse effects of metformin are gastrointestinal, including diarrhea, nausea, vomiting, and abdominal pain [6]. Reduced OCT1 activity has been associated with elevated intestinal levels of metformin, which can intensify these side effects [11,12]. These gastrointestinal reactions can negatively affect patient adherence to treatment, potentially compromising effective glycemic control. Another potential adverse effect of metformin is lactic acidosis, which results from the accumulation of lactate in the liver and muscle due to the inhibition of mitochondrial complex I [13]. Patients with impaired renal function are at a higher risk of developing lactic acidosis as reduced kidney clearance can exacerbate lactate accumulation. For this reason, metformin is contraindicated in patients with a glomerular filtration rate (GFR) below 30 mL/min per body surface area [14].

Metformin exerts its hypoglycemic effects by targeting various tissues, including the liver, skeletal muscle, and intestine. Metformin is transported into hepatocytes via OCT1 in the liver, inhibiting complex I of the mitochondrial electron transport chain [4]. This inhibition results in decreased ATP production and elevated AMP levels, activating AMP-activated protein kinase (AMPK), a key enzyme in regulating energy homeostasis. The activation of AMPK suppresses hepatic gluconeogenesis by inhibiting enzymes such as adenylate cyclase and fructose-1,6-bisphosphatase (FBP1). Furthermore, AMPK downregulates the expression of sterol regulatory element-binding protein-1C (SREBP-1C), thereby reducing the synthesis of fatty acids and minimizing fat accumulation in the liver [15]. In the intestine, metformin enhances glucagon-like peptide 1 (GLP-1) secretion through the AMPK signaling pathway, contributing to improved glucose regulation. In skeletal muscle, the activation of AMPK facilitates the translocation of GLUT4 glucose transporters to the cell membrane, promoting glucose uptake into muscle cells. This increase in glucose utilization helps to lower plasma glucose levels, further supporting its antidiabetic action [15].

### 2.1. Metabolic Actions of Metformin in the Liver

Metformin reaches high concentrations in the liver, and this tissue has been considered its central site of action for many years [16]. Metformin reduces hepatic gluconeogenesis by decreasing the respiratory chain’s mitochondrial complex I activity and reducing oxygen consumption [17,18]. The energetic imbalance produced by metformin blunted energy-consuming processes like glucose production. In rat primary hepatocytes, metformin activates AMPK, reduces the activity of its downstream target acetyl-CoA carboxylase (ACC), and stimulates fatty acid oxidation [19]. However, some recent evidence questioned the role of the AMPK pathway in metformin-induced gluconeogenesis inhibition [20]. Miller et al. propose that metformin antagonizes glucagon-induced hepatic glucose production via inhibition of PKA signaling in vitro and in vivo, in a mechanism independent of AMPK [21]. On the other hand, Madiraju et al. demonstrated that acute and chronic metformin treatment increases the cytosolic and reduces the mitochondrial redox state in the liver of Sprague-Dawley rats through non-competitive inhibition of mitochondrial glycerophosphate dehydrogenase (mGDP) [22]. The reduction in mGDP activity alters the use of glycerol and lactate as substrates for gluconeogenic flux [22]. Moreover, Duca et al. propose an interorgan axis that regulates hepatic glucose production that involves duodenal AMPK-dependent PKA activation, afferent signaling to the nucleus of the solitary tract (NTS) in the brain, and vagal stimulation of hepatic gluconeogenesis, revealing a complex system of glucose production regulation [23]. It has been proposed that metformin also regulates metabolism by modulating insulin signaling. Gunton et al. showed that metformin increases insulin receptor phosphorylation, insulin receptor substrate 2 (IRS-2) activation, GLUT1 translocation to the plasma membrane, and glucose uptake in the Huh7 cell line and primary human hepatocytes [24].

### 2.2. Metabolic Actions of Metformin in Skeletal Muscle

Skeletal muscle is the most abundant tissue in the human body, accounting for approximately 40% of body weight [25]. This tissue is the main responsible for glucose disposal in postprandial conditions, mainly in response to insulin [25]. In pathological conditions like obesity and type 2 diabetes, skeletal muscle insulin sensitivity is blunted, reducing its capacity to incorporate glucose and contributing to hyperglycemia [26]. Metformin enters muscle cells via organic cation transporter 3 (OCT3), although the intracellular concentrations achieved in muscle are lower than those achieved in the liver or kidney [26]. Regarding the metformin mechanism of action, Pavlovic et al. argued that the drug inhibits mitochondrial complex I and activates AMPK only in supra-therapeutic concentrations in C2C12 skeletal muscle cell line [27]. Conversely, Suwa et al. have demonstrated that the acute administration of metformin in rats increases AMPK phosphorylation at 6 h, while the chronic administration of metformin (14 days) increases the expression of PGC-1 alpha, cytochrome C and augments the activity of oxidative enzymes, suggesting that metformin stimulates the mitochondrial biogenesis in rat skeletal muscle [28]. Also, the oral administration of metformin for 2 weeks increases insulin-stimulated glucose uptake in the soleus muscle of mice, in a process dependent on AMPK and independent of changes in the insulin signaling pathway [29]. In a clinical context, it has been described that 10 weeks of metformin treatment in type 2 diabetic patients increases the activity and phosphorylation of AMPK at Thr-172 residue and reduces the activity of acetyl-CoA carboxylase (ACC-2) in skeletal muscle. The enhanced activity of AMPK is related to a decrease in ATP and phospho-creatine levels in diabetic patients treated with metformin [30]. Moreover, Polianskyte-Prause et al. showed that metformin potentiates insulin-induced GLUT4 translocation to plasma membrane and glucose uptake in L6 myotubes through the direct inhibition of Src homology 2 containing inositol 5-phosphatase 2 (SHIP-2) [31]. This enzyme dephosphorylates phosphatidylinositol 3,4,5-trisphosphate (PI(3,4,5)P3) to produce PI(3,4)P2, decreasing insulin-dependent signaling in plasma membrane [32]. Increased expression of SHIP-2 has been observed in insulin resistance and diabetes [32]. Concerning the actions of metformin in lipid metabolism, it has been demonstrated that metformin modifies the intramyocellular lipid profile, which plays an essential role in the development of insulin resistance in skeletal muscle [33]. Using [U-^13^C] palmitate and mass spectroscopy, Zabielski et al. showed that a high-fat diet increases intramyocellular levels of long-chain fatty acids, diacylglycerols, and ceramides in mice skeletal muscle [33]. In this context, the administration of metformin reduces the intracellular levels of these lipid mediators and improves systemic and muscular insulin sensitivity [33].

### 2.3. Metabolic Actions of Metformin in Adipose Tissue

The OCT3 transporter is expressed in adipocytes, possibly allowing metformin cellular transport [34]. Interestingly, metformin accumulates into brown adipose tissue (BAT), reaching levels comparable to the liver, kidney, and intestine [34]. Metformin stimulates glucose uptake in human adipocytes via AMPK-dependent GLUT4 translocation to the plasma membrane without affecting insulin signaling [35]. In 3T3-preadipocytes, Lee et al. showed that GLUT4 translocation to the plasma membrane depends on AMPK and Cbl/CAP signaling [36]. Metformin also regulates glucose metabolism by inhibiting the lipid phosphatase PTEN, which alters insulin signaling pathways in NIH3T3-preadipocytes [37]. Karise et al. evaluated the effect of metformin supplementation in C57Bl/6 mice fed with a high fructose diet [38]. Interestingly, metformin increases proliferation and differentiation markers in brown adipocytes, possibly through an AMPK-dependent mechanism [38]. Also, metformin induces mitochondrial biogenesis and thermogenesis and stimulates lipolysis and fatty acid uptake in BAT [38]. Moreover, Geerling et al. showed that metformin reduces total plasma cholesterol and triglycerides mainly by increasing VLDL triglyceride uptake in BAT, intracellular lipolysis, and mitochondrial fatty acid oxidation in mice [39]. Metformin treatment also reduces visceral fat mass in rats, which is related to increased fatty acid oxidation and UCP-1 overexpression in BAT [40].

### 2.4. Metabolic Actions of Metformin at the Intestinal Level

Recent works have shown that metformin reaches a high concentration in enterocytes, and its intestinal actions could play an essential role in systemic metabolic regulation [41,42]. Diverse reports indicate that metformin reduces the intestinal absorption of diet-administered glucose [43,44,45]. Zubiaga et al. [42] propose that metformin reduces the apical density of sodium-glucose transporter 1 (SGLT1), explaining the diminished glucose absorption [42]. This work also shows that the direct administration of metformin in the jejunum of minipigs increases the release of the intestinal hormone GLP-1 [42]. Consistent with these findings, Borg et al. have shown that the administration of metformin in the proximal and distal portions of the intestine reduces the plasma glucose, slows the gastric emptying, and increases the secretion of GLP-1 in type 2 diabetic patients [44]. On the other hand, it has been proposed that metformin stimulates plasma glucose transport into the enterocytes, helping to reduce plasma glucose levels in diabetic patients [45]. Tobar et al. demonstrate that metformin stimulates the basolateral transport of glucose into enterocytes in hyperglycemia through a mechanism that involves an AMPK-dependent increase in GLUT1 and GLUT2 glucose transporter expression [46]. Glucose in enterocytes is converted to lactate, reducing the portal vein’s pH and NaHCO3 levels. These processes decrease hepatic gluconeogenesis by inhibiting pyruvate carboxylase in the liver [46]. In the same direction, Schommers et al. observed that the beneficial effects of metformin in glycemic control and body weight in high-fat diet-fed mice are related to an accumulation of lactate in the intestinal wall and the venous portal circulation [47]. These authors hypothesized that the use of lactate as a substrate for hepatic gluconeogenesis generates a futile cycle of glucose-lactate-glucose, which has a high energetic cost and would explain, along with the energetic cost associated with maintaining pH homeostasis, the reduction in body weight and the reduction of insulin resistance in HFD feed mice treated with metformin [47].

## 3. Adipogenesis and Metabolic Health: Regulation, Implications, and the Role of Metformin

### 3.1. Mechanisms of Adipogenesis

Adipogenesis is the differentiation process by which a mesenchymal stem cell (MSC) develops into a functional adipocyte with specific phenotypic features. During this process, MSCs respond to extracellular signals and undergo stages of proliferation and clonal expansion that produce pre-adipocytes, cells with high plasticity that later differentiate into mature adipocytes [48,49]. Adipocytes are spherical in appearance, between 10 and 100 µm in diameter, with clustered organelles and a nucleus displaced towards the periphery of the cell due to a unilocular triglyceride vesicle that occupies most of the cytoplasm, limiting the presence of other organelles such as mitochondria and the Golgi apparatus [50,51].

Adipogenesis occurs in several well-defined stages, starting with the conversion of MSCs into preadipocytes. Although these preadipocytes are not morphologically differentiated from their progenitor cells, they show the activation of specific transcription factors. AP-1 family factors and C/EBPβ and C/EBPδ are activated in this first phase. These proteins induce a crucial second step in activating key differentiation genes, such as PPARγ and C/EBPα, which regulate adipocyte maturation and function [52,53].

PPARγ is considered the most essential transcription factor in adipogenic differentiation, as its activation induces both morphological changes and the expression of genes specific to mature adipocytes [54]. This factor is critical in white and brown adipose tissue and exists in two isoforms produced by alternative splicing. The PPARγ2 isoform is predominantly expressed in adipose tissue, promotes triglyceride storage, and is associated with obesity, insulin resistance, and dyslipidaemia. In contrast, the PPARγ1 isoform is found in several cell types and is more widely distributed [55]. The positive feedback between PPARγ and C/EBPα creates a loop that maintains sustained expression of genes essential for adipogenesis, including those involved in insulin sensitivity, lipolysis, and lipogenesis [54,56].

Adipocyte differentiation is a complex process tightly regulated by the temporal and specific expression of proteins and transcription factors. A thorough understanding of the molecular mechanisms that regulate adipogenesis is fundamental to understanding the pathogenesis of metabolic disorders such as metabolic syndrome, diabetes, and cardiovascular disease.

Adipogenesis influences the maintenance of metabolic health, particularly the metabolic processes associated with obesity. Understanding the mechanisms and regulators of adipogenesis is essential for developing effective strategies to improve metabolic health. Adipogenesis may counteract the detrimental metabolic effects of overweight and obesity by generating new adipocytes rather than expanding existing adipocytes, which tend to have a pro-inflammatory and hypoxic profile. The expansion of adipose tissue through adipogenesis results in a healthier profile characterised by smaller and more numerous adipocytes, with less inflammation and fibrosis, contributing to better metabolic health [57,58,59,60,61].

Multiple factors and events regulate adipogenesis and may contribute to the aetiology of obesity [62]. Recent evidence suggests that metformin, a hypoglycaemic agent, is emerging as a modulator of adipose tissue physiology, particularly adipogenesis. Here, we explore this topic in more detail by examining the mechanisms by which metformin may influence adipocyte differentiation and function.

### 3.2. Effect of Metformin on Adipogenesis

#### 3.2.1. Metformin’s Biphasic Role in Adipogenesis: Dose-Dependent Effects and Mechanistic Insights

The first studies that have demonstrated the effect of metformin on differentiation into mature adipocytes have been carried out mainly in experimental models using cell cultures of the 3T3 L1 mouse line or human adipose tissue pre-adipocytes.

Among the first studies reported in scientific literature on the effect of metformin on adipogenesis is the work of the group of Alexandre [63]. This work is of interest because its methodological approach is close to that currently used to achieve MSC differentiation into adipocytes. In addition, the authors considered two antecedents to establish their hypothesis. The first background was that it had already been shown that metformin can stimulate triglyceride catabolism in adipocytes through activation of adenosine monophosphate-activated protein kinase (AMPK) [64], The second was that a synthetic AMPK activator, 5-aminoimidazole 4-carboxamide ribofuranoside (AICAR), inhibits adipogenesis in the preadipocyte cell line 3T3-L1 [63].

Alexandre [63] cultured 3T3-L1 cells for 8 days exposed to 1 mM AICAR in the presence of 2–16 mM metformin. These experiments found that preadipocytes in the presence of metformin showed increased phosphorylation of AMPK at Thr172 and decreased accumulation of significantly less lipid than in untreated cells, indirectly suggesting that metformin inhibits adipogenesis. By examining the role of metformin in adipogenesis and its influence on AMPK activation, the study contributes to understanding the molecular mechanisms underlying metabolic regulation and potential targets for drug development.

Metformin affects adipogenesis through both AMPK-dependent and AMPK-independent mechanisms. At higher concentrations, metformin activates AMPK, which suppresses adipogenesis by decreasing the phosphorylation of extracellular signal-regulated kinases (ERK) and Akt while increasing the phosphorylation of p38 and c-Jun N-terminal kinase (JNK). In addition to its AMPK-mediated effects, metformin also exerts AMPK-independent effects. For example, it inhibits mTOR/p70S6K signalling, which appears to occur independently of AMPK activation. These diverse mechanisms suggest that metformin’s effects on adipogenesis involve multiple signaling pathways beyond AMPK activation [65].

Metformin has a dual effect on adipogenesis, with lower concentrations (1.25 and 2.5 mM) promoting adipocyte differentiation and higher concentrations (5 and 10 mM) inhibiting this process. This biphasic response was evident in both gene expression and lipid accumulation in the cells. Metformin significantly increased the expression of key adipogenic and lipogenic genes at lower concentrations, including PPARγ, C/EBPα, FASN, FATCD36, SCD-1, and aP2. In contrast, higher concentrations decreased the expression of these genes, highlighting the dose-dependent nature of metformin’s effects on adipogenesis. Only higher concentrations of metformin induced the phosphorylation of AMPK, p38, and JNK while reducing the phosphorylation of ERK and Akt. This suggests that the inhibitory effects of metformin on adipogenesis at higher doses may be mediated through the activation of AMPK. In support of this, pretreatment with compound C, a specific AMPK inhibitor, reversed the inhibitory effects of high doses of metformin on adipogenesis, suggesting that the anti-adipogenic effects of metformin at higher doses are at least partially dependent on AMPK signalling [66].

#### 3.2.2. Metformin’s Regulatory Effects on Adipogenesis: Modulation of Lipid Storage, Inflammation, and Epigenetic Mechanisms

Yang’s study [67] identified significant effects of metformin on adipose-derived stem cells (ADSCs), highlighting its potential to modulate fat storage and obesity-related mechanisms. One of the key findings was metformin’s ability to inhibit lipid accumulation in epididymal (Epi-ADSCs) and inguinal (Ing-ADSCs) adipose-derived stem cells, suggesting that this drug could effectively reduce fat storage.

In addition, the study showed that metformin inhibits the fusion and growth of lipid droplets in ADSCs. Consistently, metformin was found to down-regulate several proteins essential for adipogenesis, including Cidec, Perilipin1, and Rab8a. Cidec and Perilipin1 are involved in the fusion, growth, and stability of lipid droplets, while Rab8a is involved in lipid transport within cells. The reduced expression of these proteins is consistent with the inhibitory effect of metformin on lipid storage, suggesting that metformin interferes with pathways critical for the maturation and storage capacity of adipose cells. Activation of the AMPK pathway appears to be a key mediator of these effects, as it is associated with decreased expression of proteins such as Cidec, Perilipin1, and Rab8a. As this pathway is an essential regulator of energy balance and metabolism, the effect of metformin on lipid metabolism could be partly explained by its impact on AMPK signalling. The study also assessed cell viability using cell counting assay 8 (CCK-8) and showed a dose-dependent effect. While metformin concentrations of 1 mM, 2 mM, and 4 mM did not significantly affect cell viability, a concentration of 8 mM showed reduced cell viability. This suggests that the beneficial effects of metformin on lipid inhibition and adipogenesis regulation are maintained at moderate doses without compromising cell health [67].

Metformin can significantly affect the morphology, physiology, and gene expression of 3T3-L1 cells as they differentiate into adipocytes. Morphologically, metformin attenuates changes typical of adipogenesis and visibly affects the structure of lipid droplets. This change in lipid morphology suggests that metformin may reduce lipid accumulation during adipocyte differentiation, which may contribute to its anti-obesity effects. Regarding physiological changes, metformin may not only improve the appearance of adipocytes but also alter the functional properties of lipid droplets. These physiological adjustments highlight the effect of metformin on lipid metabolism and storage dynamics in adipocytes, suggesting that its action goes beyond superficial changes and extends to functional improvements in adipocytes [68]. In addition, metformin reduces pro-inflammatory cytokine secretion and enhances adipogenic differentiation in stem cells, promoting a shift to a more metabolically active phenotype. It suppresses inflammatory markers such as IL-6 and MCP-1 [69] and increases autophagy in ADSCs, reinforcing their role in regulating inflammation during adipogenesis [70].

A study by Liao [71] investigated how metformin affects FTO protein methylation and m6A modification in adipocytes. The objectives included assessing the effect of metformin on FTO expression and m6A methylation levels of key cell cycle genes, particularly those involved in mitotic clonal expansion (MCE), a crucial process in which pre-adipocytes proliferate before differentiating into mature adipocytes. The results showed that metformin inhibits FTO expression, which alters m6A methylation patterns. This change affects the interaction between m6A-modified RNAs and the YTHDF2 protein, an RNA stability and turnover regulator. Inhibition of FTO by metformin and the resulting change in the m6A methylation profile disrupts YTHDF2-mediated gene regulation. This mechanism effectively blocks the ECM, preventing pre-adipocytes from completing the cell cycle steps necessary for adipogenesis.

#### 3.2.3. Metformin’s Multifaceted Effects on Adipose Tissue: Synergistic Interactions, Oxidative Stress Reduction, and Age-Related Adipogenesis

On the other hand, a study using omental adipose tissue from forty obese individuals undergoing weight loss surgery [72] investigated the effects of metformin, insulin, and the lipid peroxidation product 4-HNE on preadipocyte adipogenesis. The researchers compared preadipocytes from insulin-resistant (IR), type 2 diabetes mellitus (T2DM), and insulin-sensitive (IS) individuals and found significant differences in their adipogenic capacity. Preadipocytes from IR and T2DM individuals showed significantly altered adipogenesis compared to those from IS individuals, with increased levels of anti-adipogenic genes. This altered adipocyte development was associated with increased levels of 4-HNE, a marker of lipid peroxidation, which in turn correlated with smaller adipocyte size and increased macrophage infiltration in the adipose tissue of patients with T2DM.

In preadipocytes derived from individuals with T2DM, combined metformin and insulin treatment improved adipogenesis more effectively than metformin treatment alone, suggesting a synergistic effect between the two drugs. This improvement was associated with a reduced presence of macrophages and reduced levels of 4-HNE, an indicator of oxidative stress, suggesting that combining the two treatments may alleviate some of the inflammatory and oxidative stress markers in adipose tissue in people with T2DM [72]. In addition, the treatment of pre-adipocytes with 4-HNE alone was shown to reduce adipogenesis and increase cell proliferation, even in the presence of metformin. However, insulin partially counteracted these negative effects, highlighting its role in promoting cell differentiation under oxidative stress conditions. These findings suggest that the combination of metformin and insulin may promote adipogenesis by reducing oxidative stress and inflammation, which may be of therapeutic benefit in the treatment of adipose tissue dysfunction in T2DM and insulin resistance [72].

The synergistic effect of insulin and metformin was also investigated by the group of Szkudelski, Konieczna, and Szkudelska [73]. In this study, adipocytes isolated from rat epididymal adipose tissue were used to assess the combined effects of both drugs on glucose transport and lipid metabolism. When administered with insulin, the results showed that metformin significantly increased glucose transport into adipocytes, suggesting a synergistic effect that enhances glucose uptake and optimises glucose metabolism in adipocytes. In addition, metformin had a significant inhibitory effect on lipolysis, or the breakdown of fat, even when stimulated by agents such as adrenaline and dibutyryl-cAMP. This inhibition was observed both at 3 mM and 12 mM glucose concentrations and when glucose was replaced by alanine, suggesting that metformin can effectively limit lipolysis under different metabolic conditions. Regarding cellular metabolism, metformin was found to reduce lactate release in adipocytes, an effect that may be related to its influence on mitochondrial function, particularly in the electron transport chain. This reduction in lactate production suggests a mechanism by which metformin improves overall metabolic efficiency, optimizing cellular energy balance and reducing the by-products of anaerobic metabolism.

Another study investigated the effects of metformin on brown adipose tissue (BAT) in a high-fructose diet model, focusing on markers of BAT proliferation, differentiation, and thermogenesis. To this end, an isoenergetic mouse model was used for ten weeks, receiving either a control diet (C) or a high fructose diet (F). For a further eight weeks, the animals were treated with metformin hydrochloride (M, 250 mg/kg/day) or saline. Results showed no significant differences in body weight gain, white fat pads, or adiposity index between the groups. Energy intake was reduced in group F, and energy expenditure was lower in groups F and FM. Metformin treatment resulted in increased BAT mass in the CM and FM groups, associated with increased adipocyte proliferation -β1-adrenergic receptor, proliferating cell nuclear antigen, and vascular endothelial growth factor- and differentiation (PR domain containing 16, bone morphogenetic protein 7), partly through activation of AMPK. In addition, metformin increased thermogenic markers in BAT through adrenergic stimulation and fibroblast growth factor 21. Metformin can also increase mitochondrial biogenesis in BAT, lipolysis, and fatty acid uptake [38].

Aging is linked to central fat redistribution and insulin resistance. To investigate age-related changes in fat tissue, Le Pelletier’s group [74] examined the senescence and adipogenic potential of adipose-derived stromal cells (ASCs) from abdominal subcutaneous fat in healthy young women (under 25 years) and older women (over 60 years). As cell passages increased, ASCs from young donors (in vitro aging) developed senescence without oxidative stress. Adipocytes derived from these ASCs exhibited impaired adipogenesis but maintained early mitochondrial function. By contrast, early-passage ASCs from older donors displayed oxidative stress and mild senescence, with derived adipocytes showing both oxidative stress and early mitochondrial dysfunction, though adipogenesis remained intact. Extended in vitro aging of ASCs from older donors led to increased senescence, mitochondrial dysfunction, oxidative stress, and severe adipocyte dysfunction. Metformin treatment did not mitigate these effects in aged ASCs from young donors; however, it did reduce oxidative stress, mitochondrial dysfunction, and senescence in ASCs from older donors. This reduction of oxidative stress and senescence by metformin improved adipogenic capacity and insulin sensitivity, mediated through AMPK activation, as confirmed by specific inhibition and activation studies. Overall, adipocytes derived from aged ASCs showed impaired adipogenesis and insulin sensitivity, suggesting that targeting stress-induced senescence in ASCs with metformin may counteract age-related adipose tissue dysfunction [74].

At a concentration of 3 mM, metformin inhibits human umbilical cord-mesenchymal stem cells (UC-MSC) proliferation and colony formation. Concurrently, it enhances their adipogenic differentiation by increasing PPARγ expression, a key regulator in the adipogenesis pathway, while downregulating FABP4 expression, which plays a role in fatty acid binding and adipocyte function. Additionally, metformin appears to exert anti-inflammatory effects, evidenced by decreased expression levels of pro-inflammatory cytokines such as IL-6, MCP-1, and COX-2 [69]. Table 1 summarizes the different cell models utilized to investigate the effects of metformin on adipogenesis. These models include both murine and human-derived preadipocytes.

Bioinformatics tools allow cross-referencing with databases of known genes and pathways, such as the AMPK pathway, which is frequently implicated in adipogenesis and is regulated by metformin. This integrated approach facilitates the robust identification of both up- and down-regulated genes and reveals potential targets that mediate the effects of metformin on lipid metabolism, inflammation, and cell differentiation. At the molecular level, high-throughput sequencing analysis revealed significant changes in the expression of adipogenesis-related genes, providing a detailed transcriptomic profile of the effect of metformin [68]. This change in the transcriptome is key to understanding how metformin affects adipocyte differentiation, involving different pathways and regulatory mechanisms (see Table 2).

### 3.3. Effect of Metformin and Vitamin D on Adipogenesis

**Figure 1:**
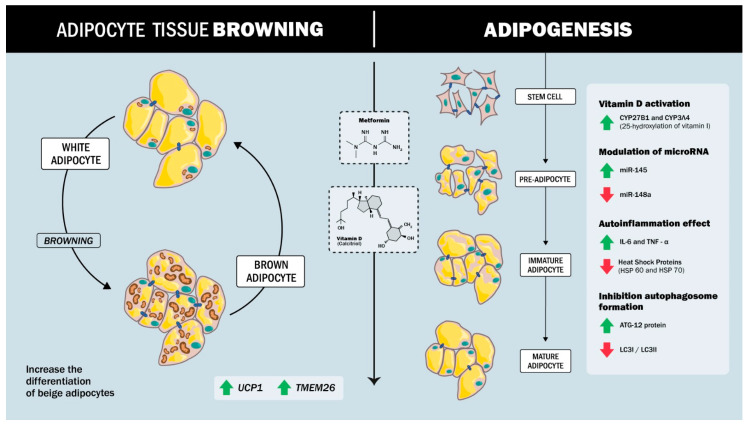
Synergistic effect between metformin and vitamin D in regulating adipogenesis and inflammation. Metformin and Vitamin D promote a shift toward a metabolically active beige adipocyte phenotype and inhibit conventional white adipogenesis, inflammation, and autophagosome formation.

The combined effect of metformin and vitamin D has been extensively studied in various experimental models and clinical trials. These studies suggest a synergistic effect between the two substances under different physiological conditions, enhancing their effect on the body (Figure 1).

The synergistic regulation of adipogenesis by vitamin D and metformin involves complex molecular mechanisms that modulate stem cell differentiation and inflammatory responses. Both agents act on ADSCs, promoting a shift toward a beige adipocyte phenotype and inhibiting conventional adipogenesis. In the studies by Cruciani’s [75], both metformin and vitamin D were found to significantly reduce adipogenesis in ADSCs, demonstrating that these substances inhibit adipocyte formation and promote differentiation into beige adipocytes. Analysis of key markers of adipogenesis, such as aP2, LPL, and ACOT2, together with the thermogenic protein UCP1 and the beige adipocyte-specific marker TMEM26, showed that metformin and vitamin D increased the expression of these markers and supported the transition to a beige phenotype [75]. Previous studies by the same group had already shown that metformin inhibits adipogenesis in the presence of vitamin D. This research suggests that metformin modulates vitamin D metabolism through the expression of specific enzymes, in particular the cytochrome P450 isoforms CYP27B1 and CYP3A4, responsible for vitamin D metabolism, and in particular 25-hydroxylation (CYP3A4 and CYP27A1). This modulation is fundamental as it suggests a regulatory mechanism that could influence the response of ADSCs to adipogenic stimuli in the presence of metformin or vitamin D.

On the other hand, miRNAs have recently been found to be involved in several metabolic dysfunctions, especially in adipose tissue, where they control processes such as adipogenesis, insulin resistance, and inflammation. For example, miR-145 is downregulated during adipogenesis, and its overexpression inhibits this process by reducing the activity of PI3K/Akt and MAPK signalling pathways [76,77]. In contrast, miR-148a promotes adipogenic differentiation when up-regulated, while its down-regulation inhibits preadipocyte differentiation [78,79,80]. In a study by Cruciani [81], it was shown that, during adipogenesis in ADSCs isolated from subcutaneous adipose tissue of human patients (men and women), miR-145 is up-regulated, whereas miR-148a is down-regulated in the presence of metformin and vitamin D, with a synergistic effect between the two compounds.

Vitamin D, particularly in its active form 1,25-dihydroxyvitamin D3, is crucial in modulating inflammation and metabolic processes in adipose tissue. Some studies have shown that this active form of vitamin D can significantly reduce pro-inflammatory markers such as interleukin-6 (IL-6), monocyte chemoattractant protein-1 (MCP-1), and tumor necrosis factor-alpha (TNF-α) in adipocytes [82,83]. These anti-inflammatory effects are mediated by the vitamin D receptor (VDR), to which vitamin D binds to inhibit NF-κB signalling, a pathway often implicated in chronic inflammation. By blocking NF-κB, vitamin D also reduces the expression of specific microRNAs closely associated with inflammatory responses [83]. In addition to its immunomodulatory effects, vitamin D promotes glucose uptake and increases AKT phosphorylation, which are key steps in metabolism and cellular energy management. This dual role—suppressing inflammation and improving metabolic function—suggests that vitamin D may play an integrative role in reducing inflammation-induced metabolic dysfunction [82].

The combination of vitamin D and metformin has shown a synergistic effect in modulating the inflammatory response and promoting the differentiation of white adipocytes to a beige phenotype. This combination therapy attenuates inflammation by targeting specific pathways that decrease pro-inflammatory cytokines while activating anti-inflammatory mechanisms [75].

A study by Cruciani [81] explored the effects of metformin and vitamin D on inflammation, autophagy, and cellular responses to stress during ADSC differentiation. The findings indicate that metformin and vitamin D, both individually and in combination, modulate adipogenic differentiation, suggesting that these compounds may influence the process of ADSC to adipocyte conversion. In terms of inflammation, the study observed a marked reduction in the secretion of pro-inflammatory cytokines, including IL-6 and TNF-α, during ADSC differentiation. This anti-inflammatory effect is particularly relevant as chronic inflammation is linked to obesity and insulin resistance. The results suggest that metformin and vitamin D may help mitigate these risk factors during cell differentiation.

Autophagy, a process essential for cellular homeostasis, was also assessed using key proteins such as ATG12, LC3B I, and LC3B II by western blot analysis. The results indicated that metformin and vitamin D treatment promoted autophagy, crucial for maintaining cell integrity and function during differentiation [81].

In addition, the treatments modulated the expression of heat shock proteins (HSPs), which are involved in the cellular stress response, as reflected by changes in HSP60 and HSP70 mRNA levels. This effect suggests that metformin and vitamin D not only reduce inflammation and autophagy but also enhance resistance to cellular stress during ADSC differentiation. Taken together, these results highlight the multifaceted role of metformin and vitamin D in regulating key processes that may contribute to the development of healthier adipocytes and improved metabolic health [81].

## 4. Conclusions

Metformin affects adipogenesis via several molecular processes, altering essential signaling and gene expression pathways. This medication, used as part of the treatment of T2DM, even in the early stages of the disease (pre-diabetes or insulin resistance), also could moderate lipid metabolism and adipocyte differentiation.

Metformin activates the AMPK pathway, which is crucial in the homeostatic control of energy balance and lipid and glucose metabolism. AMPK activation involves the inhibition of lipid droplet fusion and growth during adipogenesis through the down-regulation of proteins such as Cidec, Perilipin1, and Rab8a [67].

Metformin alters the adipocyte transcriptome, reversing the expression patterns of genes associated with adipogenesis and thus preventing lipid accumulation [68].

It also inhibits the expression of FTO, a regulator of adipogenesis. This increases the m6A methylation levels of cyclin D1 and cyclin-dependent kinase 2, reducing protein levels and blocking mitotic clonal expansion [71].

Additionally, metformin reduces the production of inflammatory cytokines in adipose tissue. Maintaining an anti-inflammatory state is relevant to mitigate insulin resistance and promote healthier adipocyte function [69].

Furthermore, there is a synergistic effect of metformin and vitamin D, where both substances influence adipogenesis through different but complementary molecular mechanisms, mainly involving the modulation of gene expression, inflammatory responses, and lipid metabolism. Both metformin and vitamin D can promote differentiation of preadipocytes toward a beige adipocyte phenotype, which is associated with increased thermogenesis, reduced lipid accumulation, and increased numbers of mitochondria.

Although metformin shows promise in combating obesity and regulating adipogenesis, some studies suggest that its effects may vary depending on individual metabolic conditions and other factors influencing adipose tissue physiology.

## Figures and Tables

**Table 1:** Cell models used to study the influence of metformin during adipogenesis.

Cells	Concentration of Metformin	Regulation on Adipogenesis	Ref.
**3T3-L1**	2–16 mM	Downregulated	[63]
	1.25 and 2.5 mM 5 mM	Upregulated Downregulated	[65]
	2.5, 5 and 10 mM	Downregulated	[68]
**C3H10T1/2**	0.1 mM to 10 mM	Downregulated	[64]
	500 μM	Downregulated	[65]
**ADSCs (adipose-derived stem cells)** **Epi-ADSCs and Ing-ADSCs**	2 mM and 4 mM	Downregulated	[67]
**3T3-L1**	5 mM	Downregulated	[68]
**ADSC** **of men and women (age = 45 ± 15 years, BMI: 22 ± 3 kg/m^2^)**	5 mM	Downregulated	[70]
**UC-MSC** **(human umbilical cord-mesenchymal stem cells)**	3 mM	Upregulated	[69]
**Stromal vascular fraction-derived preadipocytes from obese diabetic patients**	1 mM	Upregulated	[72]

**Table 2:** Key genes involved in adipogenesis, their primary functions, and regulatory changes in response to metformin.

Gene	Function	Regulation	Ref.
**PPARγ**	Master regulator of adipogenesis; activates lipid uptake and storage	Downregulated	[68]
**C/EBPα**	Works with PPARγ to regulate differentiation and lipid metabolism	Downregulated	
**FABP4**	Facilitates fatty acid transport and intracellular lipid storage	Downregulated	
**SCD1**	Involved in fatty acid synthesis and desaturation	Downregulated	
**LPL**	Catalyzes hydrolysis of triglycerides in lipoproteins	Downregulated	
**CD36**	Fatty acid transporter involved in lipid uptake and storage	Downregulated	
**ACSL1**	Activates long-chain fatty acids for lipid synthesis and storage	Downregulated	
**G0S2**	Regulates lipolysis and fatty acid storage in adipocytes	Downregulated	
**PDE3B**	Modulates cAMP levels affecting lipolysis and adipocyte differentiation	Downregulated	
**ABCG1**	Mediates cholesterol and lipid efflux from cells	Downregulated	
**LEP (Leptin)**	Involved in energy regulation and satiety signaling	Downregulated	
**AMPK**	Central energy regulator, enhances catabolic processes	Activated	
**FTO**	Regulates m6A RNA methylation, impacting adipogenesis through gene expression stability	Downregulated	
**KLF4**	Promotes early adipocyte differentiation	Downregulated	
**Cidec**	Interacts with the AMPKα1 subunit, leading to its degradation via the ubiquitin-proteasome pathway. This interaction promotes adipocyte differentiation by reducing AMPKα levels.	Downregulated	[67]
**Perilipin1**	Coat lipid droplets in adipocytes, protect them from lipolysis, and facilitate triglyceride storage. PKA phosphorylates it during lipolytic stimulation, which enhances lipolysis by hormone-sensitive lipase and adipose triglyceride lipase.	Downregulated	
**Rab8a**	Pivotal regulator in adipogenesis, influencing both Wnt signaling attenuation and lipid droplet fusion.	Downregulated	
